# Recommendations on Weight Loss and Healthy Lifestyle in Prostate Cancer Clinical Guidelines: A Systematic Review

**DOI:** 10.3390/ijerph19031452

**Published:** 2022-01-27

**Authors:** Mario Rivera-Izquierdo, Virginia Martínez-Ruiz, José Juan Jiménez-Moleón

**Affiliations:** 1 Departamento de Medicina Preventiva y Salud Pública, Universidad de Granada, 18016 Granada, Spain; virmruiz@ugr.es (V.M.-R.); jjmoleon@ugr.es (J.J.J.-M.); 2 Service of Preventive Medicine and Public Health, Hospital Universitario San Cecilio, 18016 Granada, Spain; 3 Instituto de Investigación Biosanitaria ibs.Granada, 18012 Granada, Spain; 4 Centro de Investigación Biomédica en Red de Epidemiología y Salud Pública (CIBERESP), 28029 Madrid, Spain

**Keywords:** clinical guidelines, consensus statement, prostate cancer, obesity, mortality, body weight

## Abstract

Obesity is associated with negative prostate cancer outcomes (e.g., specific mortality, all-cause mortality, biochemical recurrence, etc.), according to the current scientific literature. Nevertheless, recommendations on weight loss and healthy lifestyles are poorly covered by clinicians. We aimed at identifying these recommendations from clinical practice guidelines (CPGs) for prostate cancer. We systematically searched MEDLINE, EMBASE, Web of Science, Scopus, guideline databases and online sources for CPGs updated from January 2015 to August 2021. The searches were independently conducted by two researchers, without language restrictions. A total of 97 prostate cancer guidelines, including 84 (86.6%) CPGs and 13 (13.4%) consensus statements, were included. Recommendations on reaching and maintaining a healthy weight or healthy lifestyles were provided by 7 (7.2%) and 13 (13.4%) documents, respectively. No differences regarding recommendations were found by type of document, year of publication or country. Our results suggest that professional societies and governments should update prostate cancer guidelines to include these recommendations for improving prostate cancer prognosis.

## 1. Introduction

Prostate cancer (PC) is the most frequent cancer in men [1]. Nevertheless, few prognostic factors have been clearly associated with PC outcomes, mainly older age, ethnicity and family history of PC [2], none of them modifiable. For decades, obesity has been associated with negative PC outcomes, although results were not always consistent [3,4]. According to the PRACTICAL consortium [5] and the REDUCE study [6], among others, obesity may be considered a modifiable risk factor for prostate cancer according to current data. Recently, new studies on the molecular mechanisms linking obesity to prostate cancer have been developed [7], and several systematic reviews and meta-analyses have pointed to obesity, measured as body mass index (BMI) ≥ 30 kg/m^2^, as a prognostic factor associated with a higher frequency of prostate cancer specific mortality [8], higher frequency of all-cause mortality [8] and higher frequency of biochemical recurrence after radical prostatectomy [9]. The World Cancer Research Fund [10] also reported an increased risk of being diagnosed with advanced PC in obese patients. Other authors have reported the increased difficulties of prostate cancer surgery in obese patients, which can lead to adverse events or disease recurrence [11], and a higher association with the need for chemotherapy [12]. Other recent studies have pointed to an association between higher BMI and multiple pelvic lymph node metastasis [13].

Given that obesity is associated with poorer health outcomes in the general population [14,15], and the wide range of works reporting its implications for negative PC outcomes, it seems logical to include recommendations on weight loss from clinicians to newly diagnosed PC patients. Therefore, several authors have pointed out the need for including recommendations on reaching and maintaining a healthy weight in clinical practice guidelines (CPGs), as weight loss programs have proven to be effective [16,17]. In fact, current research is focused on designing healthy lifestyle interventions and weight loss programs [18,19]. CPGs are documents that compile current evidence-based recommendations on how to diagnose and treat a medical condition, usually endorsed by medical organizations or governments. Consensus statements (CSs) are a comprehensive summary of the opinions of an expert panel to provide guidance on controversial or poorly understood aspects of healthcare. Therefore, both documents should include updated evidence-based information on modifiable prognostic factors such as weight loss or lifestyle habits.

The aim of this study was to compile all recent CPGs and CSs on prostate cancer diagnosis and treatment developed by professional societies or governments and to analyze the presence of recommendations regarding healthy weight and lifestyles.

## 2. Materials and Methods

The systematic review was reported according to the Preferred Reporting Items for Systematic Reviews and Meta-Analyses (PRISMA) [20] (Supplementary Table S1).

### 2.1. Search Strategy and Data Source

We conducted a systematic search covering the period from January 2015 to August 2021 to avoid selection bias, as most of the guidelines before this period had been updated and replaced by more recent ones, combining MeSH terms “clinical practice guidelines”, “guidelines”, “consensus”, “prostate cancer”, “prostate cancer diagnosis” and “prostate cancer treatment” and including word variants in the TRIP database and MEDLINE, without language restrictions, to collect all updated CPGs and CSs. When the language of the document was not the mother tongue of the researchers (i.e., different from English or Spanish), we tried to contact researchers fluent in this language from our center. When this was not possible, we completely translated the document using free translator software, DeepL (https://www.deepl.com/translator, accessed on: 15 December 2021).

Afterwards, we extended the search to other databases, such as EMBASE, Web of Science, Scopus, Cochrane Database of Systematic Reviews and the ACP Journal Club. Eight guideline databases were searched, including the National Institute for Health and Care Excellence (NICE), the National Comprehensive Cancer Network (NCCN), the Scottish Intercollegiate Guidelines Network (SIGN), Fisterra, the Canadian Clinical Practice Guideline (CPG), the CMA Infobase, the National Health and Medical Research Council (NHMRC), the Health Services Technology Assessment Texts (HSTAT) and the Guidelines International Network (GIN). Finally, 77 professional society websites were visited to complete the search (Supplementary Table S2), and references from other relevant studies were manually searched.

### 2.2. Study Selection and Data Extraction

We covered CPGs and CSs on the diagnosis and therapeutic management of prostate cancer, developed by professional societies, organizations or government agencies. We also included guidelines on the management of cancer complications (e.g., castration-resistant prostate cancer). The documents were considered as CPGs and CSs as depicted by the authors after full-text reading, or when the search database considered it as so. The presence of keywords in the title, such as “guideline” for CPGs or “consensus” for CSs, helped us in the classification of the documents. Obsolete documents updated in more recent years from the same organization or government, CPGs or CSs for education or information purposes and documents designed only for patients were excluded. Two independent reviewers (MR-I and VM-R) identified titles and abstracts and performed full-text assessment of the eligible studies. Disagreements or inconsistences were solved by consensus with a third senior reviewer (JJJ-M). Duplicate documents were identified and removed. Data extraction and identification of duplicates were conducted using the software Mendeley Reference Manager version 2.61.1. (Mendeley, London, UK).

### 2.3. Assessment

All CPGs and CSs were thoroughly assessed for the inclusion of recommendations concerning weight loss or lifestyle. The variables collected from each document were the type of document (CPG or CS), focus of the document (diagnostic or treatment of PC), area (divided by continent and country), year of the last update and publication in a journal. The variables of interest were divided into the following groups: (1) acknowledgement of obesity, weight or body mass index (BMI) as a potential risk factor for PC; (2) acknowledgement of lifestyle as a risk factor for PC; (3) acknowledgement of obesity, weight or BMI as possible prognostic factors for PC; (4) acknowledgement of lifestyle as a prognostic factor for PC; (5) recommendations on healthy weight for PC patients; (6) recommendations on healthy lifestyle for PC patients; (7) recommendations on healthy diet for PC patients and (8) recommendations on physical activity for PC patients. Finally, a quality assessment of CPGs that included such recommendations was conducted using the AGRE-II tool. For points (1) to (5), we searched the guideline text for an appropriate statement that included the presence of body weight or lifestyle as recognized risk or prognostic factors for PC, after full-text reading. For points (6) to (8), we looked for specific recommendations provided in the document. All the selected documents were assessed independently by two reviewers (MR-I and VM-R), and disagreements were resolved by consensus with a third reviewer (JJJ-M).

### 2.4. Statistical Analyses

We conducted descriptive analysis on the presence of obesity, weight, BMI or lifestyle in the guidelines. Country, year, type of document and focus of the document were considered for bivariate analyses. Differences in the presence of the variables of interest were analyzed using T-tests and chi-square tests for quantitative and qualitative variables, respectively. When chi-square conditions for applications were not met, Fisher’s exact test was applied.

## 3. Results

### 3.1. Study Selection

**Figure 1:**
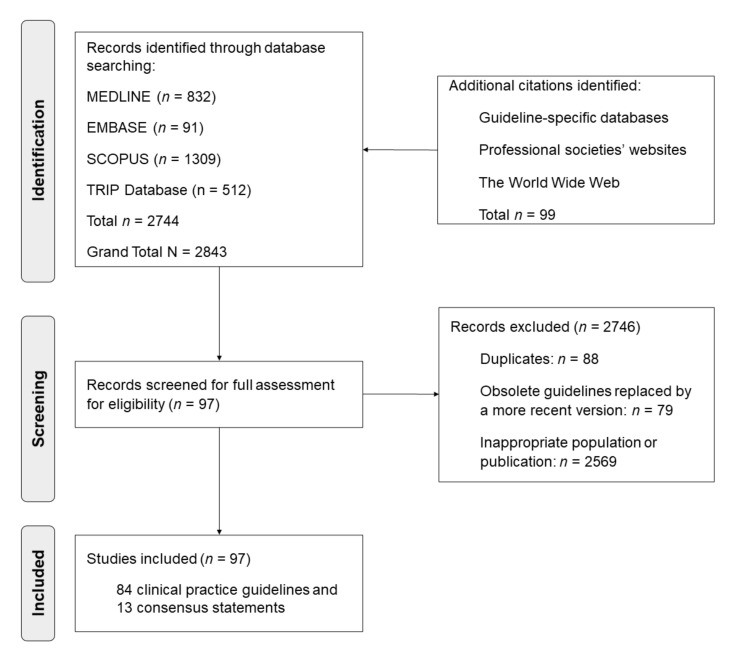
Flow chart of the study selection of the systematic review according to PRISMA guidelines.

Of the 2905 identified citations, 97 CPGs and CSs met the inclusion criteria; 45 were (46.5%) published in a journal [21,22,23,24,25,26,27,28,29,30,31,32,33,34,35,36,37,38,39,40,41,42,43,44,45,46,47,48,49,50,51,52,53,54,55,56,57,58,59,60,61,62,63,64,65] and 52 (53.6%) were published in other sources (Supplementary Table S3). Figure 1 shows the flow chart of the study selection.

### 3.2. Characteristics of the Studies

Table 1 shows the main characteristics of the selected documents, including the title, year and country. There was a total of 40 (41.2%) North American guidelines, 35 (36.5%) European guidelines, 12 (12.4%) Asian guidelines, 6 (6.2%) South American guidelines and 4 (4.1%) from other continents (African, Oceanian or international guidelines that covered countries from different continents). From the selected documents, 84 (86.6%) were CPGs and 13 (13.4%) were CSs. A total of 45 (46.4%) documents corresponded to diagnostic guidelines and 78 (80.4%) included information on therapeutic approaches and recommendations.

### 3.3. Factors Associated with Recommendations on Obesity and Healthy Lifestyles

Only 11 (11.3%) documents acknowledged obesity, body mass index or weight as a risk factor for prostate cancer, and five (5.2%) as a prognostic factor. Similarly, 15 (15.5%) documents considered lifestyle factors as risk factors, and seven (7.2%) as prognostic factors. Regarding recommendations, only seven (7.2%) guidelines provided advice on reaching or maintaining a healthy weight for PC patients, and 13 (13.4%) provided advice on healthy lifestyles. These 13 documents that presented recommendations showed reasonably high quality according to AGREE-II (Supplementary Table S4). Supplementary Table S5 shows examples of the specific recommendations provided by these 13 documents. Specifically, healthy diet and physical activity advice was provided in 9 (9.3%) and 10 (10.3%) documents, respectively (Table 2).

Table 3 shows the different characteristics of the guidelines when comparing the 13 documents that provided recommendations on healthy lifestyles with the 84 documents that did not. Although no significant differences were found between the two subgroups, a tendency to provide more recommendations was observed in more recent documents, in CPGs compared with CSs and in therapeutic guidelines. Nevertheless, recommendations on healthy lifestyles were very infrequent for all subgroups

## 4. Discussion

We present a thorough systematic review including CPGs and CSs regarding prostate cancer diagnosis and treatment from 2015 to 2021 with no language restrictions. We found that acknowledgment and recommendations on healthy weight and lifestyle for PC patients were very infrequent, regardless of the type of document, year of publication and country. We surprisingly found a high quantity of guidelines (*n* = 97), most of them on the same topics, suggesting that there may exist a redundancy in prostate cancer guidelines.

Several studies have proved that obesity and other lifestyle factors, such as healthy diet or physical exercise, are risk factors for a diagnosis of PC [66] and prognostic factors once the diagnosis is established [8,9]. Therefore, the World Cancer Research Fund [10] and the World Health Organization [67] have included obesity as an important factor to be controlled for improving PC risk or prognosis. Importantly, PC-specific mortality, all-cause mortality and biochemical recurrence have been reported to be increased in obese patients [8,9]; therefore, current studies are focused on testing programs for reaching and maintaining a healthy weight after PC diagnosis [16,17,18,19]. There are also multiple agencies, including the Prostate Cancer Foundation, Mayo Clinic and multiple patient advocacy groups, that make recommendations on healthy lifestyles for PC patients. Nevertheless, as proven in this study, PC guidelines throughout the world poorly cover recommendations on this important aspect. As healthy lifestyles also improve different outcomes such as cardiovascular events [68], chronic diseases [69] and overall survival [8], it seems evident that recommendations on this topic should be reinforced from clinicians and official guidelines provided for all PC patients.

Specifically, guideline developers should include appropriate professionals (e.g., nutritional therapists or experts in adapted physical exercise) and patient representatives as proper members in guideline panels, to ensure that nutritional and healthy lifestyle topics are included in the prioritized guideline questions. It is also advisable to perform scoping exercises at the beginning of guideline development to make sure that all aspects of health related to PC are properly covered. Moreover, clinicians and healthcare professionals that interact with PC patients should use our data to reinforce recommendations regarding weight loss and healthy lifestyles. According to the recommendations summarized in Supplementary Table S5, PC patients should be counseled regarding the importance of modifiable health-related behaviors or risk factors, such as smoking and obesity, through healthy diet, toxic habits and physical exercise assessment and counselling [32]. This is especially recommended, according to evidence-based data, for patients undergoing androgen-deprivation therapies [33] and for patients that receive surgical treatment (i.e., prostatectomy), where obesity has been more clearly associated with poorer prognosis [32].

It is important to note that different recommendations might be individualized according to the disease stage. For example, behavioral interventions regarding vegetable consumption have not been proven to decrease cancer progression at early stages [70]. In this regard, several clinical trials are being developed to analyze the impact of weight loss interventions in patients with clinically localized PC [71]. A recent systematic review on the MARTINI-Lifestyle cohort also pointed out that the adherence to lifestyle recommendations is very poor in PC patients [72]. However, this adherence has been proven to reduce mortality in several types of cancer [73]. For PC, preclinical and observational studies have identified potential benefits for high-vegetable, low-fat, low-meat diets and increased exercise, but Level I evidence is still limited [74]. Therefore, randomized clinical trials are still needed to inform specific recommendations for PC patients, considering the stage of the disease and the most appropriate intervention.

Another important aspect for future research is to add information on the perspectives and values of patients through qualitative research, to optimize the design of healthy lifestyle interventions that better adhere to patients’ possibilities and perspectives.

A key strength of our study was the global perspective, including guidelines from all countries included in our search, with almost one hundred documents included. No restriction to specific languages, data sources or types of documents were considered. For documents not written in English or Spanish (mother tongue of the researchers), we attempted to contact a pertinent researcher in our center, but, when this was not possible, we used free translation software. This could imply a limitation given that the translation software may present mistakes. Another perceived limitation of the study was the difficulties in finding documents with languages different from English, French, Spanish, German and Chinese. We tried to minimize this issue by duplicating data extraction through two independent reviewers. An additional important limitation of this study is that no specific tool for evaluating the presence of recommendations in clinical guidelines was available; therefore, we analyzed their presence manually through extensive reading of the selected documents, and we used the tool AGREE-II for evaluating the quality of the guidelines that covered weight loss or lifestyle recommendations. We did not prospectively register the protocol of this systematic review.

We found that only a seventh of all PC guidelines recommended the adoption of healthy lifestyles and only 7.2% provided advice on reaching or maintaining a healthy weight, despite the current evidence regarding its usefulness. Our data suggest that professional societies and governments should update their guidelines and documents regarding PC and include recommendations on healthy lifestyles after diagnosis. Clinicians from Oncology, Urology and Primary Care units should advise their PC patients to reach and maintain a healthy weight through recommendations on diet and adapted physical activity, according to the patients’ preferences. Future studies should provide reflections or data on how to systematically introduce weight loss or healthy lifestyle programs for improving PC prognosis.

## 5. Conclusions

Recommendations on healthy weight or lifestyles are very infrequently provided in PC clinical guidelines from professional societies or governments, regardless of the date of publication, type of document or country. Nevertheless, the current literature indicates that healthy weight and lifestyles improve PC risk and prognosis. Future clinical trials should be developed for informing each PC patient about the best lifestyle intervention, considering the disease stage. Therefore, there may not be a standard recommendation, but different approaches depending on the patient’s disease state, lipid profile, genetics or other unknown variables. PC guidelines should be updated to cover this important issue. Future strategies or intervention programs to reach and maintain a healthy weight should be designed for improving PC care.

## Figures and Tables

**Table 1:** Clinical guidelines and consensus statements on diagnosis and treatment of prostate cancer (*n* = 97), 2015–2021.

Name of the Clinical Practice Guideline	Entity	Country	Year	Recommendation ^1^
PMB definition guideline: Prostate cancer	CMS	South Africa	2020	No
South African prostate cancer guidelines	SAUA	South Africa	2017	Yes
Chinese guidelines for diagnosis and treatment of prostate cancer 2018	NHC China	China	2018	No
Evidenced-based clinical practice guideline for prostate cancer (summary: Japanese Urological Association, 2016 edition)	JUA	Japan	2016	No
2020 Korean guidelines for the management of metastatic prostate cancer.	KSMO	Korea	2020	No
Prostate cancer	MIMS	Malaysia	2021	Yes
Singapore Cancer Network (SCAN) Guidelines for the Management of Advanced Castrate-Resistant Prostate Cancer.	SCAN	Singapore	2015	No
Saudi Oncology Society and Saudi Urology Association combined clinical management guidelines for prostate cancer 2017	SOS-SUA	Saudi Arabia	2017	No
EAU-EANM-ESTRO-ESUR-SIOG Guidelines on Prostate Cancer—2020 Update. Part 1: Screening, Diagnosis, and Local Treatment with Curative Intent	EAU-EANM-ESTRO-ESUR-SIOG	Europe	2020a	No
EAU-EANM-ESTRO-ESUR-SIOG Guidelines on Prostate Cancer. Part II-2020 Update: Treatment of Relapsing and Metastatic Prostate Cancer	EAU-EANM-ESTRO-ESUR-SIOG	Europe	2020b	Yes
Biochemical recurrence in prostate cancer: The EAU Prostate Cancer Guidelines Panel’s recommendations.	EAU-EANM-ESTRO-ESUR-SIOG	Europe	2020c	No
ESMO Clinical Practice Guidelines for diagnosis, treatment and follow-up of prostate cancer	ESMO	Europe	2020	Yes
Guidelines on Prostate Cancer	EAU-ESTRO-ESOR-SIOG	Europe	2018	No
EAU-ESTRO-SIOG Guidelines on prostate cancer: screening, diagnosis and local treatment with curative intent	EAU-ESTRO-SIOG	Europe	2017	No
DUCG’s National Guidelines for Diagnosis and Treatment of Prostate Cancer	DUCG	Denmark	2015	No
French ccAFU guidelines—update 2020–2022: prostate cancer	CCAFU	France	2020	No
S3—Prostate cancer guideline	AWMF-DKG-DKH	Germany	2021	Yes
PSMA ligand PET/CT in the diagnosis of prostate carcinoma.	AWMF	Germany	2019	No
National Prostate Cancer GP Referral Guideline	NCCP	Ireland	2018	No
Diagnosis, staging and treatment of patients with prostate cancer. National Clinical Guideline No. 8	NCCP	Ireland	2016	Yes
Prostate cancer, national guideline version 3.0	IKNL	Netherlands	2017	Yes
Appropriate use of pharmaceutical products for patients with castration-refractory prostate cancer	Zorginstituut Nederland	Netherlands	2016	No
Prostate cancer	NVU	Netherlands	2016	No
SEOM clinical guidelines for the treatment of advanced prostate cancer (2020)	SEOM	Spain	2020	No
SEOM clinical guidelines for the treatment of metastatic prostate cancer (2017)	SEOM	Spain	2017	No
Enzalutamide for treating hormone-sensitive metastatic prostate cancer (technology appraisal guidance TA712)	NICE	UK	2021	No
Darolutamide with androgen deprivation therapy for treating hormone-relapsed non-metastatic prostate cancer (technology appraisal guidance TA660)	NICE	UK	2020	No
Prostate cancer: diagnosis and management. (NICE guideline NG131)	NICE	UK	2019a	No
Enzalutamide for hormone-relapsed non-metastatic prostate cancer (Technology appraisal guidance TA580)	NICE	UK	2019b	No
Padeliporfin for untreated localised prostate cancer (Technology appraisal guidance TA546)	NICE	UK	2018a	No
Memokath-051 stent for ureteric obstruction (Medical technologies guidance MTG35)	NICE	UK	2018b	No
Prostate cancer screening with prostate-specific antigen (PSA) test: a clinical practice guideline	MAGIC–BMJ	UK	2018	No
Biodegradable spacer insertion to reduce rectal toxicity during radiotherapy for prostate cancer (Interventional procedures guidance IPG590)	NICE	UK	2017	No
Irreversible electroporation for treating prostate cancer Interventional procedures guidance [IPG572]	NICE	UK	2016a	No
Radium-223 dichloride for treating hormone-relapsed prostate cancer with bone metastases (Technology appraisal guidance TA412)	NICE	UK	2016b	No
Cabazitaxel for hormone-relapsed metastatic prostate cancer treated with docetaxel (Technology appraisal guidance TA391)	NICE	UK	2016c	No
Degarelix for treating advanced hormone-dependent prostate cancer (Technology appraisal guidance TA404)	NICE	UK	2016d	No
Abiraterone for castration-resistant metastatic prostate cancer previously treated with a docetaxel-containing regimen (Technology appraisal guidance TA259)	NICE	UK	2016e	No
Abiraterone for treating metastatic hormone-relapsed prostate cancer before chemotherapy is indicated (Technology appraisal guidance TA387)	NICE	UK	2016f	No
Enzalutamide for treating metastatic hormone-relapsed prostate cancer before chemotherapy is indicated (Technology appraisal guidance TA377)	NICE	UK	2016g	No
Suspected cancer: recognition and referral (NICE guideline NG12)	NICE	UK	2015	No
Brachytherapy for Patients with Prostate Cancer: American Society of Clinical Oncology/Cancer Care Ontario Joint Guideline Update	ASCO/CCOJ	USA/ Canada	2017	No
Canadian Urological Association guideline on androgen deprivation therapy: Adverse events and management strategies	CUA	Canada	2021a	Yes
Canadian Urological Association best practice report: Prostate-specific membrane antigen positron emission tomography/computed tomography (PSMA PET/CT) and PET/magnetic resonance (MR) in prostate cancer	CUA	Canada	2021b	No
2021 Canadian Urological Association (CUA)-Canadian Uro-Oncology Group (CUOG) guideline: Management of castration-resistant prostate cancer (CRPC)	CUA	Canada	2021c	No
Multiparametric Magnetic Resonance Imaging in the Diagnosis of Clinically Significant Prostate Cancer. Guideline 27-2 version 2	CCO	Canada	2021	No
A Canadian framework for managing prostate cancer during the COVID-19 pandemic: Recommendations from the Canadian Urologic Oncology Group and the Canadian Urological Association	CUA	Canada	2020a	No
Canadian Urological Association-Canadian Urologic Oncology Group guideline on metastatic castration-naive and castration-sensitive prostate cancer	CUA	Canada	2020b	Yes
Local prostate cancer. Clinical Practice Guideline GU-012—Version 3	CCA	Canada	2020a	No
Advanced/Metastatic prostate cancer. Clinical Practice Guideline GU-010—Version 2	CCA	Canada	2020b	No
Prostate Cancer Part 1: Diagnosis and Referral in Primary Care	BC	Canada	2020a	No
Prostate Cancer Part 2: Follow-up in Primary Care	BC	Canada	2020b	Yes
An Endorsement of the 2018 Guideline on Hypofractionated Radiation Therapy for Localized Prostate Cancer: An ASTRO, ASCO, and AUA Evidence-Based Guideline	CCO	Canada	2018	No
Guideline for Optimization of Surgical and Pathological Quality Performance for Radical Prostatectomy in Prostate Cancer Management. Evidence-Based Series 17-3 Version 2	CCO	Canada	2017a	No
Follow-up Care for Survivors of Prostate Cancer—Clinical Management: a Program in Evidence-Based Care Systematic Review and Clinical Practice Guideline	CCO	Canada	2017b	No
Canadian Urological Association recommendations on prostate cancer screening and early diagnosis	CUA	Canada	2017	No
Multiparametric magnetic resonance imaging for pre-treatment local staging of prostate cancer: A Cancer Care Ontario clinical practice guideline	CCO	Canada	2016a	No
Bone Health and Bone-Targeted Therapies for Prostate Cancer. Guideline 3-14 Version 2	CCO	Canada	2016b	Yes
Prostate cancer, 2015.	CCA	Canada	2015	No
Society for Immunotherapy of Cancer (SITC) clinical practice guideline on immunotherapy for the treatment of urothelial cancer	SITC	USA	2021	No
Initial Management of Noncastrate Advanced, Recurrent, or Metastatic Prostate Cancer: ASCO Guideline Update	ASCO	USA	2021	No
Advanced prostate cancer: AUA-ASTRO-SUO guideline	AUA-ASTRO-SUO	USA	2020	No
Bone Health and Bone-Targeted Therapies for Prostate Cancer: ASCO Endorsement of a Cancer Care Ontario Guideline	ASCO	USA	2020	No
Prostate cancer: NCCN Clinical Practice Guidelines in Oncology	NCCN	USA	2019a	No
Prostate cancer early detection. NCCN Clinical Practice Guidelines in Oncology	NCCN	USA	2019b	No
Incontinence after Prostate Treatment: AUA/SUFU Guideline (2019)	AUA-SUFU	USA	2019	No
Adjuvant and Salvage Radiotherapy after Prostatectomy: ASTRO/AUA Guideline	ASTRO-AUA	USA	2019	No
Prostate cancer prevention and early detection.	ACS	USA	2019	No
Castration-resistant prostate cancer	AUA	USA	2018	No
Screening for Prostate Cancer: US Preventive Services Task Force Recommendation Statement	USPSTF	USA	2018	No
Early detection of prostate cancer: AUA guideline	AUA	USA	2018	No
Clinically Localized Prostate Cancer: ASCO Clinical Practice Guideline Endorsement	ASCO	USA	2018	Yes
ASTRO/ASCO/AUA Guideline on Hypofractionation for Localized Prostate Cancer	ASTRO-ASCO-AUA	USA	2018	No
American Joint Committee on Cancer. Prostate	AJCC	USA	2017	No
Clinically Localized Prostate Cancer: AUA-ASTRO-SUO Guideline.	AUA-ASTRO-SUO	USA	2017	Yes
NCCN Clinical Practice Guidelines in Oncology (NCCN Guidelines). Version 3.	NCCN	USA	2016	No
Radiotherapy for recurrent prostate cancer: 2018 Recommendations of the Australian and New Zealand Radiation Oncology Genito-Urinary group	FROGG	Australia and New Zealand	2018	No
Clinical practice guidelines: PSA Testing and Early Management of Test-Detected Prostate Cancer	PCFA	Australia and New Zealand	2016	No
AUGE Clinical Guidelines. Prostate cancer in patients over 15 years old.	MSC	Chile	2015	No
Prostate cancer. Risk factors, early detection and PSA: screening, use and correct interpretation	AMUC	Costa Rica	2018	No
Prostate cancer diagnosis and treatment. Clinical practice guidelines.	IMSS	Mexico	2018	No
Clinical practice guideline: prostate cancer	AUNA	Peru	2019	No
Clinical practice guideline for the screening, diagnosis and treatment of localized and locally advanced prostate cancer	IETSI	Peru	2021	No
Clinical Practice Guideline for the early detection, diagnosis, staging, treatment, rehabilitation and follow-up of patients with prostate cancer.	INEN	Peru	2021	No
**Name of the Consensus Statement**	**Entity**	**Country**	**Year**	**Recommendation ^1^**
Update of Guidelines for Management of Prostate Cancer in West Africa 2019: Consensus Working Document.	WA	West Africa	2019	No
NCCN Asia Consensus Statement prostate cancer	NCCN	Asia	2018	No
Chinese Expert Consensus on the Diagnosis and Treatment of Castration-Resistant Prostate Cancer (2019 Update)	CEC	China	2019	No
Consensus statements on the management of clinically localized prostate cancer from the Hong Kong Urological Association and the Hong Kong Society of Uro-Oncology	HKUA-HKSUO	China	2019	No
Expert Group Consensus Opinion on Prostate Cancer Diagnosis and Management in India	Consensus	India	2020	No
Guidance for the assessment and management of prostate cancer treatment induced bone loss. A consensus position statement from an expert group	Expert group	UK	2020	Yes
Canadian consensus forum of key controversial areas in the management of advanced prostate cancer	GURC	Canada	2021	No
Current topics in radiotherapy for genitourinary cancers: Consensus statements of the Genitourinary Radiation Oncologists of Canada	GUROC	Canada	2020	No
Canadian consensus algorithm for erectile rehabilitation following prostate cancer treatment	CUA	Canada	2018	No
Cancer Care Ontario Position Statement on Prostate Cancer Screening using the Prostate-Specific Antigen (PSA) Test	CCO	Canada	2017	No
Second-Line Hormonal Therapy for Men with Chemotherapy-Naïve, Castration-Resistant Prostate Cancer: ASCO Provisional Clinical Opinion	ASCO	USA	2017	No
Role of Genetic Testing for Inherited Prostate Cancer Risk: Philadelphia Prostate Cancer Consensus Conference 2017.	PPCCC	USA	2017	No
Management of patients with advanced prostate cancer: APCCC consensus conference	APCCC	International	2019	No

The guidelines are presented divided by type of document (CPGs or CSs), continent, country and year. The complete names of the entities (abbreviations) are available in Supplementary Table S2. ^1^ Presence of any recommendation regarding body weight or lifestyle in the document.

**Table 2:** Characteristics of the clinical practice guidelines (CPGs) and consensus statements (CSs) on prostate cancer regarding assessment or recommendations on obesity and healthy lifestyles.

Characteristics	N	%
Total sample	97	100.0
Obesity, body mass index or weight are considered as risk factors for prostate cancer in the guideline	11	11.3
Lifestyle factors are considered as risk factors for prostate cancer in the guideline	15	15.5
Obesity, body mass index or weight are considered as prognostic factors for prostate cancer in the guideline	5	5.2
Lifestyle factors are considered as prognostic factors for prostate cancer in the guideline	7	7.2
Recommendations on reaching or maintaining a healthy weight are provided within the guideline	7	7.2
Recommendations on reaching or maintaining healthy lifestyle habits are provided within the guideline	13	13.4
Recommendations on healthy diet are provided within the guideline	9	9.3
Recommendations on physical activity are provided within the guideline	10	10.3

**Table 3:** Characteristics of the clinical practice guidelines (CPGs) and consensus statements (CSs) stratified by the presence of recommendations on obesity or healthy lifestyle.

Characteristics	Total (*n* = 97)	Presence of Recommendations (*n* = 13)	Absence of Recommendations (*n* = 84)	*p*-Value ^1^
	N (%)	N (%)	N (%)	
**Year of publication**				0.668
Published in 2018 or after	35 (36.1)	9 (14.5)	53 (85.5)	
Published before 2018	62 (63.9)	4 (11.4)	31 (88.6)	
**Type of document**				0.689
CPGs	84 (86.6)	12 (14.3)	72 (85.7)	
CSs	13 (13.4)	1 (7.7)	12 (92.3)	
**Continent**				
European guidelines	35 (36.1)	6 (17.1)	29 (82.9)	0.537
North American guidelines	40 (41.2)	5 (12.5)	35 (87.5)	0.827
South American guidelines	6 (6.2)	0 (0.0)	6 (100.0)	0.594
Asian guidelines	12 (12.4)	2 (16.7)	10 (83.3)	0.723
**Publication in a journal**				
Published in a journal	45 (46.4)	6 (13.3)	39 (86.7)	1.000
Not published in a journal	52 (53.6)	7 (13.5)	45 (86.5)	
**Focus of the guideline ^2^**				
Diagnostic guidelines	45 (46.4)	6 (13.3)	39 (86.7)	0.985
Therapeutic guidelines	78 (80.4)	12 (15.4)	66 (84.6)	0.246

^1^*p*-value of chi-square test or Fisher’s exact test, when appropriate. ^2^ Diagnostic and treatment guidelines account for more than 100% as several documents were both diagnostic and treatment guidelines. Bold: distinguish between groups of variables.

## Data Availability

All datasets used for this study are presented in the manuscript and its Supplementary Materials.

## Supplementary Materials

The following supporting information can be downloaded at: https://www.mdpi.com/article/10.3390/ijerph19031452/s1, Table S1: PRISMA 2020 Checklist, Table S2: Data sources and search strategy, Table S3: Identified clinical guidelines and consensus statements not published in a journal. Table S4. Appraisal of Guidelines for Research & Evaluation (AGREE-II) assessment of clinical practice guidelines on prostate cancer that complied with recommendations for healthy weight or lifestyle habits (n = 13). Table S5. Examples of recommendations regarding healthy weight or lifestyles in clinical practice guidelines of prostate cancer.
