# Supplementary material for: Recommendations on Weight Loss and Healthy Lifestyle in Prostate Cancer Clinical Guidelines: A Systematic Review
Source: Int J Environ Res Public Health. 2022 Jan 27;19(3):1452. doi: 10.3390/ijerph19031452 (PMC8835487; doi:10.3390/ijerph19031452)
Supplement: Supplementary file 1 [file ijerph-19-01452-s001.zip › Table S2.pdf]

Supplementary Table S2.

Data sources and search strategy.

**Sample search strategy for Medline**
#1 Practice guideline [pt]<br>#2 Practice guidelines as topic [mesh]<br>#3 Guideline [pt]<br>#4 guidelines as topic [mesh]<br>#5 consensus [mesh]<br>#6 OR #1-#5<br>#7 prostate neoplasms [mesh]<br>#8 prostate neoplasms [all]<br>#9 prostate cancer [mesh]<br>#10 prostate cancer [all]<br>#10 OR #7-9<br>#11 2015 [pdta] : 3000[pdta]<br># #6 AND # 10 AND #11
**Online databases**
1. MEDLINE<br>2. EMBASE<br>3. Web of Science<br>4. Scopus<br>5. The Cochrane Database of Systematic Reviews<br>6. Cochrane Methodology Register<br>7. ACP Journal Club<br>8. Database of Abstracts of Reviews of Effects<br>9. Cochrane Central Register of Controlled Trials (CENTRAL)<br>10. The Health Technology Assessment
**Guideline-specific databases**
1. National Health and Medical Research Council (NHMRC), Australia<br>2. Canadian Medical Association (CMA) Infobase, Canada<br>3. Clinical Practice Guidelines (CPG) Infobase, Canada<br>4. New Zealand Guidelines Group (NZGG), New Zealand<br>5. National Institute for Health and Care Excellence (NICE), UK<br>6. Trip Database, UK<br>7. Scottish Intercollegiate Guidelines Network (SIGN), UK<br>8. Fisterra, Spain<br>9. Heath Services and Technology Assessment Texts (HSTAT), USA<br>10. National Comprehensive Cancer Network (NCCN), USA
**Professional societies' websites**
1. Academia Nacional de Medicina (ANM), Argentina<br>2. Australian Government, Australia<br>3. Faculty of Radiation Oncology Genito-urinary group (FROGG), Australia & New Zealand<br>4. Prostate Cancer Foundation of Australia (PCFA), Australia & New Zealand<br>5. Belgian Healthcare Knowledge Centre (KCE), Belgium<br>6. Brazilian Society of Surgical Oncology (BSSO), Brazil<br>7. Alberta Health Services, Canada<br>8. CancerCare Manitoba, Canada<br>9. Cancer Care Ontario (CCO) Canada<br>10. Ontario Ministry of Health, Canada<br>11. Genito-urinary radiation oncologists of Canada (GUROC), Canada<br>12. Ministerio de Salud de Chile, Chile<br>13. Chinese expert consensus meeting, China<br>14. Chinese Ministry of Health, China<br>15. National Health Commission (NHC), China

16. Hong Kong Urological Association (HKUA), China
17. Hong Kong Society of Uro-Oncology (HKSUO), China
18. Instituto Nacional de Cancerología, Colombia
19. Dirección de desarrollo de Servicio de Salud, Costa Rica
20. Asociación de Médicos Urólogos Costarricenses (AMUC), Costa Rica
21. Danish Prostate Cancer Database (DUCG), Denmark
22. European Association of Urology (EAU), Europe
23. European Association of Nuclear Medicine (EANM), Europe
24. European Society of Medical Oncology (ESMO), Europe
25. European School of Oncology (ESO), Europe
26. European School of Radiology (ESOR), Europe
27. European Society of Pathology (ESP), Europe
28. European Society of Radiation Oncology (ESTRO), Europe
29. European Society of Urogenital Radiology (ESUR), Europe
30. International Society of Geriatric Oncology (SIOG), Europe
31. St. Gallen/Vienna, Europe
32. Cancer Committee of the French Urological Association (CCAFU), France
33. Association of the Scientific Medical Societies in Germany (AWMF), Germany
34. German Cancer Aid (DKH), Germany
35. German Cancer Society (DKG), Germany
36. Urological Society of India (USI), India
37. Indian Cooperative Oncology Network (ICON), India
38. Expert group consensus opinion, India
39. Advanced Prostate Cancer Consensus Conference (APCCC), International
40. Enhanced Recovery After Surgery (ERAS), International
41. International expert panel, International
42. International Society of Urological Pathology (ISUP)
43. National Cancer Control Programme (NCCP), Ireland
44. Japanese Urological Association (JUA), Japan
45. Korean Society of Medical Oncology (KSMO), Korea
46. Lithuanian oncologist, endocrinologist and General practitioners, Lithuania
47. Medical Malaysian Multi-channel (MIMS), Malaysia
48. Instituto Mexicano del Seguro Social (IMSS), Mexico
49. Netherlands Comprehensive Cancer Organisation (IKNL)
50. Dutch Urological Association (NVU), Netherlands
51. Richtlijnendatabase, Netherlands
52. Ministry of Health from New Zealand, New Zealand
53. Instituto de Evaluación de Tecnologías en Salud e Investigación (IETSI), Peru
54. Instituto Nacional de Enfermedades Neoplásicas (INEN), Peru
55. Saudi Oncology Society (SOS), Saudi Arabia
56. Saudi Urology Society (SUA), Saudi Arabia
57. Compliance Association & Network (SCAN), Singapore
58. South African Urological Association (SAUA), South Africa
59. Federación de Sociedades Españolas de Oncología (FESEO), Spain
60. Sociedad Española de Oncología Médica (SEOM), Spain
61. British Association of Urological Surgeons (BAUS), UK
62. Joint Guidelines from British Surgical Associations, UK
63. National Health Service, UK
64. The Royal College of Pathologists, UK
65. The Royal College of Radiologists, UK
66. Royal College of Radiologists (RCR), UK
67. Scottish Cancer Taskforce, UK
68. American Board of Internal Medicine's, USA
69. American Brachytherapy Society, USA
70. American Society of Plastic Surgeons, USA
71. American Society for Radiation Oncology, USA
72. American Cancer Society (ACS), USA

73. American Medical Society (AMS), USA
74. American Society of Clinical Oncology (ASCO), USA
75. American Society for Radiation Oncology (ASTRO), USA
76. American Urological Association (AUA), USA
77. Society of Surgical Oncology (SSO), USA

The professional societies are presented divided by alphabetical order of their country.
