# Supplementary material for: Recommendations on Weight Loss and Healthy Lifestyle in Prostate Cancer Clinical Guidelines: A Systematic Review
Source: Int J Environ Res Public Health. 2022 Jan 27;19(3):1452. doi: 10.3390/ijerph19031452 (PMC8835487; doi:10.3390/ijerph19031452)
Supplement: Supplementary file 1 [file ijerph-19-01452-s001.zip › Table S3.pdf]

Supplementary Table S3.

Identified clinical practice guidelines and consensus statements not published in a journal.

(CMS). Council for Medical Schemes. PMB definition guideline: Prostate cancer. 2020.
(SAUA). South African Urological Association. South African prostate cancer guidelines. 2017.
(MIMS). Malaysian multi-channel provider of drug information, medical education and services. Prostate Cancer. 2021.
(EAU-EANM-ESTRO-ESUR-SIOG). EAU-EANM-ESTRO-ESUR-SIOG. Biochemical recurrence in prostate cancer: The EAU Prostate Cancer Guidelines Panel's recommendations. 2020.
(EAU-ESTRO-ESOR-SIOG). EAU-ESTRO-ESOR-SIOG Guidelines on prostate cancer. 2018.
(DUCG). DUCG's National Guidelines for Diagnosis and Treatment of Prostate Cancer, 2015.
(AWMF-DKG-DKH). AWMF-DKG-DKH. S3 prostate cancer guideline. 2021.
(AWMF). AWMF. PSMA ligand PET/CT in the diagnosis of prostate carcinoma. 2019.
(NCCP). NCCP. National Prostate Cancer GP Referral Guideline. 2018.
(NCCP). NCCP. Diagnosis, staging and treatment of patients with prostate cancer. National Clinical Guideline No. 8. 2016.
(NCCN). Prostate cancer early detection. NCCN Clinical Practice Guidelines in Oncology. 2019.
(IKNL). IKNL. Prostate cancer, national guideline version 3.0. 2017.
(Zoginstituut Netherlands). Zorginstituut Netherlands. Appropriate use of pharmaceutical products for patients with castration-refractory prostate cancer. 2016.
(NVU). NVU. Prostate cancer. 2016.
AUA-ASTRO-SUO. Advanced prostate cancer: AUA-ASTRO-SUO guideline. 2020.
(NICE). The National Institute for Health and Care Excellence. Enzalutamide for treating hormone-sensitive metastatic prostate cancer (technology appraisal guidance TA712). 2021.
(NICE). The National Institute for Health and Care Excellence. Darolutamide with androgen deprivation therapy for treating hormone-relapsed non-metastatic prostate cancer (technology appraisal guidance TA660). 2020.
(NICE). The National Institute for Health and Care Excellence. Prostate cancer: diagnosis and management. (NICE guideline NG131). 2019.
(NICE). The National Institute for Health and Care Excellence. Enzalutamide for hormone-relapsed non-metastatic prostate cancer (Technology appraisal guidance TA580). 2019.
(NICE). The National Institute for Health and Care Excellence. Padeliporfin for untreated localised prostate cancer (Technology appraisal guidance TA546). 2018.
(NICE). The National Institute for Health and Care Excellence. Memokath-051 stent for ureteric obstruction (Medical technologies guidance MTG35). 2018.
(NICE). The National Institute for Health and Care Excellence. Biodegradable spacer insertion to reduce rectal toxicity during radiotherapy for prostate cancer (Interventional procedures guidance IPG590). 2017
(NICE). The National Institute for Health and Care Excellence. Irreversible electroporation for treating prostate cancer. Interventional procedures guidance [IPG572]. 2016.
(NICE). The National Institute for Health and Care Excellence. Radium-223 dichloride for treating hormone-relapsed prostate cancer with bone metastases (Technology appraisal guidance TA412. 2016.
(NICE). The National Institute for Health and Care Excellence. Cabazitaxel for hormone-relapsed metastatic prostate cancer treated with docetaxel (Technology appraisal guidance TA391). 2016.
(NICE). The National Institute for Health and Care Excellence. Degarelix for treating advanced hormone-dependent prostate cancer (Technology appraisal guidance TA404). 2016.

(NICE). The National Institute for Health and Care Excellence. Abiraterone for castration-resistant metastatic prostate cancer previously treated with a docetaxel-containing regimen (Technology appraisal guidance TA259). 2016.
(NICE). The National Institute for Health and Care Excellence. Abiraterone for treating metastatic hormone-relapsed prostate cancer before chemotherapy is indicated (Technology appraisal guidance TA387). 2016.
(NICE). The National Institute for Health and Care Excellence. Enzalutamide for treating metastatic hormone-relapsed prostate cancer before chemotherapy is indicated (Technology appraisal guidance TA377). 2016.
(NICE). The National Institute for Health and Care Excellence. Suspected cancer: recognition and referral (NICE guideline NG12). 2015.
(CCO). CCO. Multiparametric Magnetic Resonance Imaging in the Diagnosis of Clinically Significant Prostate Cancer. Guideline 27-2 version 2. 2021.
(CCA). Cancer Care Alberta. Local prostate cancer. Clinical Practice Guideline GU-012 – Version 3. 2020.
(CCA). Cancer Care Alberta. Advanced/Metastatic prostate cancer. Clinical Practice Guideline GU-010 – Version 2. 2020.
(BC). British Columbia. Prostate Cancer Part 1: Diagnosis and Referral in Primary Care. 2020.
(BC). British Columbia. Prostate Cancer Part 2: Follow-up in Primary Care. 2020.
(CCO). Cancer Care Ontario. An Endorsement of the 2018 Guideline on Hypofractionated Radiation Therapy for Localized Prostate Cancer: An ASTRO, ASCO, and AUA Evidence-Based Guideline. 2018.
(CCO). Cancer Care Ontario. Guideline for Optimization of Surgical and Pathological Quality Performance for Radical Prostatectomy in Prostate Cancer Management. Evidence-Based Series 17-3 Version 2. 2017.
(CCO). Cancer Care Ontario. Cancer Care Ontario Position Statement on Prostate Cancer Screening using the Prostate-Specific Antigen (PSA) Test. 2017.
(CCO). Cancer Care Ontario. Bone Health and Bone-Targeted Therapies for Prostate Cancer. Guideline 3-14 Version 2. 2016.
(CCA). Cancer Control Alberta. Prostate cancer. 2015.
(ACS). ACS. Prostate cancer prevention and early detection. 2019.
(AUA). AUA. Castration-resistant prostate cancer Cookson. 2019.
(AUA). AUA. Early detection of prostate cancer: AUA guideline. 2018
(AUA-ASTRO-SUO). Clinically Localized Prostate Cancer: AUA-ASTRO-SUO Guideline. 2017.
(NCCN). NCCN Clinical Practice Guidelines in Oncology (NCCN Guidelines). Version 3. 2016.
(PCFA). PCFA. Clinical practice guidelines: PSA Testing and Early Management of Test-Detected Prostate Cancer. 2016.
(MSC). MSC. AUGE Clinical Guidelines. Prostate cancer in patients over 15 years old. 2015.
(AMUC). AMUC. Prostate cancer. Risk factors, early detection and PSA: screening, use and correct interpretation. 2018.
(IMSS). IMSS. Prostate cancer diagnosis and treatment. Clinical practice guidelines. 2018.
(AUNA). AUNA. Clinical practice guideline: prostate cancer. 2019.
(IETSI). IETSI. Clinical practice guideline for the screening, diagnosis and treatment of localized and locally advanced prostate cancer. 2021.
(INEN). INEN. Clinical Practice Guideline for the early detection, diagnosis, staging, treatment, rehabilitation and follow-up of patients with prostate cancer. 2021.
