# Supplementary material for: Recommendations on Weight Loss and Healthy Lifestyle in Prostate Cancer Clinical Guidelines: A Systematic Review
Source: Int J Environ Res Public Health. 2022 Jan 27;19(3):1452. doi: 10.3390/ijerph19031452 (PMC8835487; doi:10.3390/ijerph19031452)
Supplement: Supplementary file 1 [file ijerph-19-01452-s001.zip › Table S4.pdf]

Supplementary Table S4. Appraisal of Guidelines for Research & Evaluation (AGREE-II) assessment of clinical practice guidelines on prostate cancer that complied with recommendations for healthy weight or lifestyle habits (*n = 13)*. The complete version of the tool is available from https://agreetrust.org. The tool is composed by 23 items. Each item is assessed in a 7-point scale (from 1: strongly disagree to 7: strongly agree). Therefore, the maximum punctuation of the tool is 161 points. The percentage of the punctuation according to the maximum possible punctuation for each domain in presented in the cells. Correspondence between each guideline (Entity, year) and their complete name can be consulted in Supplementary Table S2. The guidelines are presented divided by year of the last update.

**Entity, year**	**Domain 1: Scope and purpose (items 1-3)**	**Domain 2: Stakeholder involvement (items 4-6)**	**Domain 3: Rigour of development (items 7-14)**	**Domain 4: Clarity of presentation (items 15-17)**	**Domain 5: Applicability (items 18-21) (MPP: 28)**	**Domain 6: Editorial Independence (items 22-23)**	**Total score (Global Quality)**
AWMF-DKG-DKH, 2021	85.7%	76.2%	75.0%	85.7%	71.4%	42.9%	5 / 7
CUA, 2021a	80.1%	47.6%	46.4%	90.5%	85.7%	28.6%	4 / 7
MIMS, 2021	47.6%	57.1%	44.6%	76.2%	71.4%	35.7%	3.5 / 7
BC, 2020b	80.1%	57.1%	42.8%	81.0%	67.9%	71.4%	4 / 7
CUA, 2020b	85.7%	85.7%	83.9%	81.0%	67.9%	50.0%	5.5 / 7
EAU-EANM-ESTRO-ESUR-SIOG, 2020b	80.1%	81.0%	87.5%	85.7%	71.4%	85.7%	6 / 7
ESMO, 2020	71.4%	81.0%	87.5%	85.7%	78.6%	85.7%	6 / 7
ASCO, 2018	80.1%	76.2%	83.9%	100%	67.9%	71.4%	6 / 7
ASCO-ASTRO-SUO, 2017	80.1%	81.0%	83.9%	85.7%	71.4%	71.4%	6 / 7
IKNL, 2017	42.8%	28.6%	35.7%	47.6%	60.7%	28.6%	3 / 7
SAUA, 2017	52.4%	52.4%	35.7%	28.6%	50.0%	28.6%	3 / 7
CCO, 2016b	52.4%	76.2%	92.9%	85.7%	85.7%	35.7%	5.5 / 7
NCCP, 2016	90.5%	85.7%	92.9%	90.5%	89.3%	35.7%	6.5 / 7
**Mean**	70.4%	67.1%	67.4%	78.2%	72.3%	50.0%	4.5 / 7
