# Supplementary material for: Recommendations on Weight Loss and Healthy Lifestyle in Prostate Cancer Clinical Guidelines: A Systematic Review
Source: Int J Environ Res Public Health. 2022 Jan 27;19(3):1452. doi: 10.3390/ijerph19031452 (PMC8835487; doi:10.3390/ijerph19031452)
Supplement: Supplementary file 1 [file ijerph-19-01452-s001.zip › Table S5.pdf]

Supplementary Table S5. Examples of recommendations regarding healthy weight or lifestyles in clinical practice guidelines of prostate cancer.

**Entity, year**	**Examples of recommendations**
AWMF-DKG-DKH, 2021	Recommendations.<br>“The four main recommendations of the guideline are:<br>a. Aim for a healthy weight.<br>b. Be physically active.<br>c. Be sure to eat a healthy diet with an emphasis on plant-based products.<br>d. Reduce your alcohol consumption.”
CUA, 2021a	Recommendations.<br>“Providers should obtain a comprehensive baseline physical examination prior to ADT initiation that includes blood pressure, weight, waist circumference, and calculation of body mass index (BMI)”.<br>“Management of the complications of androgen-deprivation therapy:<br>• Lifestyle changes to promote healthy diet and weight<br>• Smoking cessation<br>• Exercise therapy”
MIMS, 2021	“Advise patients on ADT to have a healthy weight and diet, stop smoking, lessen alcohol intake, meet recommended levels of calcium and vitamin D, and have an annual screening for diabetes and dyslipidemia”<br>“While further studies are needed, the results suggest that modifiable factors can mitigate the consequences of having a genetic susceptibility to prostate cancer”
BC, 2020b	*Patients with primary treatment of surgery, radiation therapy, or androgen deprivation therapy. Management options.*<br>General quality of life and psychosocial sequelae.<br>“Men should be encouraged to participate in an exercise program.<br>• Advise patients on strategies for achieving and maintaining a healthy weight using diet and exercise.<br>• During scheduled follow-up clinical visits, assess men’s psychosocial status; if distress is evident, refer to specialized care to address social and emotional quality of life, as well as support groups for coping training for couples when applicable.<br>• Use of standardized assessment tools is recommended (e.g., EPIC or PHQ9)”
CUA, 2020b	“Men with mCNPC/mCSPC treated with ADT should be encouraged to take vitamin D (1000 IU daily) and total calcium intake of at 800–1000 mg daily, and to make specific lifestyle changes, including smoking cessation, reduction in alcohol and caffeine intake, and increase in weight-bearing exercises.”

EAU-EANM-ESTRO-ESUR-SIOG, 2020b	“Prostate cancer patients should be counseled regarding the importance of modifiable health-related behaviors or risk factors, such as smoking and obesity (Expert Opinion)”<br>“While age is a well-established risk factor for prostate cancer, there are now clear data showing that other patient-related factors such as smoking and excess body weight, typically assessed as a high body mass index (BMI), are correlated with prostate cancer death. Moreover, these factors are also risk factors for death from any cause. As such, clinicians are strongly encouraged to use the time of prostate cancer diagnosis as a “teachable moment” to counsel patients about weight loss and smoking cessation. In regards to surgically treated patients, in general, smoking, older age, and obesity increase the risk of perioperative complications, including bleeding, infections, and deep venous thromboses in non-prostate surgeries. As similar results have been seen elsewhere in the urological literature, there is no reason to believe these factors do not contribute to perioperative morbidity from prostate cancer”<br>“In summary, there is strong circumstantial evidence that smoking and obesity may adversely impact treatment outcomes in men undergoing treatment for prostate cancer. Given these concerns, the Panel felt that patients should be informed of the risks. Moreover, the Panel agreed that most patients should be offered the opportunity to delay therapy for a few months to allow them time to lose weight or stop smoking to reduce these perioperative risks as long as doing so does not significantly impair cancer control”
ESMO, 2020	“Lifestyle measures to maintain bone health are recommended for men on ADT: weight-bearing exercise, stopping smoking, two or fewer units alcohol daily, adequate calcium intake and vitamin D status (reach and maintain reference vitamin D levels) [IV, B]”
ASCO, 2018	“For guideline statement 2: Prostate cancer patients should be counseled regarding the importance of modifiable health-related behaviors or risk factors, such as smoking and obesity (Expert Opinion), the panel also suggests that patients in need of counseling for smoking cessation and/or weight loss be referred to appropriate evidence-based intervention programs as warranted”
ASCO-ASTRO-SUO, 2017	“While age is a well-established risk factor for prostate cancer, there are now clear data showing that other patient-related factors such as smoking and excess body weight, typically assessed as a high body mass index (BMI), are correlated with prostate cancer death. Moreover, these factors are also risk factors for death from any cause. As such, clinicians are strongly encouraged to use the time of prostate cancer diagnosis as a “teachable moment” to counsel patients about weight loss and smoking cessation. In regards to surgically treated patients, in general, smoking, older age, and obesity increase the risk of perioperative complications, including bleeding, infections, and deep venous thromboses in non-prostate surgeries. As similar results have been seen elsewhere in the urological literature, there is no reason to believe these factors do not contribute to perioperative morbidity from prostate cancer.”
IKNL, 2017	Measures.<br>• Determine the goal of nutritional treatment.

	• Encourage the patient to exercise, preferably under the direction of an (oncology) physiotherapist. In complex situations, advice from an oncological physiotherapist, rehabilitation physician or sports physician is advisable.<br>• Check whether the advised nutrition can be used and adjust the advice if necessary.<br>• Check the weight progression and if possible the body composition.<br>• Evaluate whether the goal of the nutritional treatment is being achieved”
SAUA, 2017	Prevention.<br>“A healthy lifestyle is the backbone of prevention of the vast majority of cancers and must be given as generic advice” Prevention and treatment of adverse events due to prostate cancer treatments.<br>• Lifestyle/Exercise/Diet”
CCO, 2016b	“[…] counselling about lifestyle management and risk factor modification to reduce the risk of bone loss and falls (e.g., moderating alcohol intake, stopping smoking, optimizing calcium intake, and vitamin D supplementation)”, “At the same time, multiple studies have demonstrated important gaps in the quality of bone health care for men with prostate cancer, including low rates of BMD testing either before or while on ADT, low rates of diet and lifestyle counselling”. Research questions. “Intervention: Drugs, supplements, lifestyle modifications, exercise“
NCCP, 2016	“The main goal for the NCCP Survivorship Programme is to empower patients to achieve their best possible health while living with and beyond a diagnosis of cancer. This involves providing information, guidance and support to survivors and their families and healthcare professionals in relation to healthy lifestyle, disease prevention and control”
